# Demographic and Socioeconomic Factors Associated with Fitbit Ownership in the NIH *All of Us* Cohort

**DOI:** 10.3390/ijerph23070839

**Published:** 2026-06-26

**Authors:** Bryson Carrier, James W. Navalta

**Affiliations:** 1 Department of Natural Sciences, Oregon Institute of Technology, Klamath Falls, OR 97601, USA; 2 Department of Kinesiology and Nutrition Sciences, University of Nevada, Las Vegas, NV 89154, USA; james.navalta@unlv.edu

**Keywords:** fitness trackers, activity monitor, socioeconomic disparities, demographic disparities, gender identity, public health

## Abstract

**Highlights:**

**Public health relevance—How does this work relate to a public health issue?**

- Wearable fitness trackers are increasingly used in public health research and clinical settings to monitor physical activity, yet ownership rates vary substantially across demographic groups, leaving key populations underrepresented in wearable-derived health data.
- Using the NIH All of Us Research Program cohort (N = 633,547), this study examines how gender identity, race, ethnicity, and socioeconomic factors predict Fitbit ownership, addressing a gap in population-level characterization of wearable ownership.

**Public health significance—Why is this work of significance to public health?**

- Female and gender-diverse participants had higher odds of Fitbit ownership, while Black or African American, Native Hawaiian and Pacific Islander/Middle Eastern or North African (NHPI/MENA), and “None-Indicated” race participants had lower odds compared to White participants; Hispanic or Latino participants had higher odds than non-Hispanic participants, an association obscured by studies combining race and ethnicity.
- Interestingly, higher ZIP3-level median household income was associated with lower overall odds of Fitbit ownership, but this gradient varied significantly by race: for Black or African American participants, the relationship reversed, with higher area income predicting higher ownership.

**Public health implications—What are the key implications or messages for practitioners, policy makers and/or researchers in public health?**

- Racial and gender disparities in wearable ownership have direct implications for the representativeness of wearable-derived datasets; researchers and policymakers should account for these gaps when generalizing findings from fitness tracker data to broader populations.
- While provisioned-device programs, such as the NIH All of Us Research Program WEAR initiative, offer a model for reducing economic barriers to wearable ownership, they do not eliminate socioeconomic influences on Fitbit ownership rates. Targeted outreach and culturally informed engagement strategies remain necessary to address persistent racial disparities.

**Abstract:**

Wearable fitness trackers are increasingly popular for monitoring health-related metrics, yet their ownership patterns across socioeconomic, demographic, and gender-diverse populations remain underexplored at a population level. This study utilized data from the NIH All of Us Research Program to investigate how area-level socioeconomic status, race, and gender identity influence wearable device ownership. Methods. Data were analyzed from 633,547 participants from the All of Us Dataset. Fitbit ownership was modeled with four binary logistic regression models: a demographics-only model, a ZIP3-level socioeconomic indicators model, and a combined model incorporating four demographic × median household income interactions (race, gender, age, and Hispanic/Latino ethnicity), and an intersectional model adding a race x gender interaction. Continuous socioeconomic predictors were rescaled for interpretability (median income per USD 10,000; area-level fractions per 10 percentage points). Socioeconomic-adjusted models were restricted to 606,414 participants with available ZIP3-linked data. Fitbit ownership was defined as having a Fitbit record in the database. Results. Fitbit ownership was observed in 8.34% of the study population. Logistic regression analyses revealed significant demographic disparities: female participants and gender-diverse identities had significantly higher odds of ownership than males (OR = 1.25–2.2). Black or African American (OR = 0.38) and NHPI/MENA (OR = 0.82) participants had lower odds compared to White participants, while Asian (OR = 1.13), more than one race (OR = 1.25), and Hispanic or Latino (OR = 1.25) participants had higher odds. Each USD 10,000 increase in ZIP3 median household income was associated with 12.5% lower odds of ownership overall (OR = 0.875), but this gradient varied significantly by race. For Black or African American participants, the relationship reversed direction (OR = 1.08 per $10,000). A race x gender interaction further showed that female ownership was not uniform across race, being the largest among Black or African American participants (OR = 2.27) and reversed among Asian participants (OR = 0.87). ZIP3 socioeconomic data were structurally unavailable for all American Indian or Alaska Native participants due to the *All of Us* program’s small-population ZIP3 aggregation policy, precluding their inclusion in socioeconomic-adjusted models. Conclusions. This analysis demonstrates significant gender, racial, and socioeconomic disparities in wearable fitness tracker ownership, showing significantly higher device usage among females and gender-diverse individuals, but lower usage among certain racial groups and a seemingly contradictory negative ownership rates among higher socioeconomic levels. Ownership patterns nonetheless appear more equitable than in consumer cohorts, likely reflecting the device-provision programs undertaken by the NIH.

## 1. Introduction

Wearable technology are devices that can be worn on the body and present information enabling user interaction through voice command or physical input [1]. A subclassification of wearables is fitness trackers, which provide information about physiological measures during physical activity, such as heart rate, step count, and energy expenditure [2]. While this information can be used to make informed decisions about exercise routines, the accuracy of returned measures varies [2,3]. Since 2016, wearable technology has been listed among the top world-wide fitness trends [4,5], with Fitbit devices consistently among the most popular [6].

While the use of wearable fitness trackers has become widespread, literature on the adoption of devices and intersectional factors is limited. Nagappan et al. used data from the Rock Health Digital Health Consumer Adoption Survey consisting of 10,679 wearable owners and 13,295 non-owners [7]. People earning less than USD 25,000 USD had a smaller percentage of owners than non-owners, and people who were covered by Medicare were less likely to own wearables than people who were on private insurance or insurance from their employer [7]. Wearable ownership varied across racial and ethnic groups, with higher proportions of non-Hispanic Black, non-Hispanic Asian, and Hispanic/Latin respondents in the owner group [7]. Cheung et al. evaluated several models in the framework of people’s intention to adopt wearable technology [8]. From a sample of 171 respondents, it was reported that health belief and health information accuracy had a significant impact on perceived usefulness, which impacted the intention to adopt a wearable device [8]. Additionally, consumer innovativeness and the influence of a reference group were critical drivers of the intention to adopt [8]. It should be noted that All of Us data come from the Wearables Enhancing All of us Research (WEAR) initiative, where Fitbit devices were provided to invited participants from underrepresented communities, free of charge. They also launched a Bring-Your-Own-Device initiative, enabling participants to donate their Fitbit data (along with other data of interest) to the program [9]. From this standpoint, the All of Us Research Program (All of Us) may be the best dataset to date to evaluate the question of Fitbit ownership [10]. Thus, this study represents a population-level assessment of factors that may affect ownership of wearable fitness trackers, such as socioeconomic status, race, and gender.

Chandrasekaran et al. utilized data from the National Cancer Institute’s Health Information National Trends Survey (4551 respondents) and reported that those who were most likely to use wearable healthcare devices were women (see below), White individuals (see below), and those with an annual household income greater than USD 75,000 (2.6 times more likely than those with an income less than USD 20,000 USD) [11]. Kim et al. used the 4.0 data release from the Adolescent Brain and Cognitive Development Study (ABCD; 10,414 respondents, 7424 who enrolled in the wearable device cohort and 2990 who did not), and reported a significantly lower proportion of children were from households with an income less than USD 24,999 than in the non-wearable cohort [12]. Shandhi et al. evaluated survey responses from the Duke University Health System and reported that people with full-time employment (64%) had higher wearable device ownership than other categories (employed part time = 59%, retired, not looking for work = 52%; disabled, not able to work = 53%; not employed, but looking = 61%; not employed, not looking = 55%) [13]. Based on this emerging data, it is apparent that socioeconomic variables could have an impact on wearable fitness tracker usage.

Emerging data on the effect of race and ethnicity on wearable device engagement are less clear. Chandrasekaran et al. reported that White individuals (1.65 times more likely than non-Hispanic Asian individuals) were among those who were most likely to use wearable healthcare devices [11]. Kim et al. reported a significantly lower relative proportion of Black children (−59%) in the device cohort than the non-wearable cohort [12]. From the Duke University Health System, Shandhi et al. observed no significant differences for smart device ownership between people of different race/ethnicity profiles (White = 60%, Black = 56%, Asian = 62%, Hispanic = 67%) [13]. Due to these uncertainties in the current literature, there is a need to further explore the interactions between race and ethnicity and wearable fitness tracker usage.

Finally, the literature suggests that sex and gender may impact how people interact with wearable fitness devices. Chandrasekaran et al. reported that women (1.26 times more likely than men) were among those who were most likely to use wearable healthcare devices [11]. Similarly, Shandhi et al. utilized survey responses from the Duke University Health System to determine that females (62%) were more likely to own a wearable device compared with demographic counterparts (males = 53%) [13]. While Kim et al. did not test for gender differences among the children enrolled in the ABCD Study, some participants identified as “other” (including participants who indicated “different”, “gender queer,” or “trans” for their gender) or did not know or refused to answer [12]. Further determination of the effect of wearable device usage is needed, especially for those who belong to the gender-diverse community.

Importantly, the investigation into the ownership of wearable technology in the All of Us dataset is useful to determine if the data obtained can be applied to a broad range of sub-populations, across different socioeconomic or demographic variables, or to what sub-populations it does not sufficiently represent, and thus where it should not be applied. This is important because this dataset could provide a wealth of granular information about physical activity, sleep, and other factors measured by the devices. If this dataset has sub-populations that are not sufficiently represented, researchers and program managers should be aware of the appropriate applicability of the dataset.

It is clear that further characterization of factors driving wearable and fitness tracker usage must be explored. The purpose of this investigation was to utilize the availability of the large All of Us Research Project dataset (goal, attract 1 million individuals) to determine which factors, such as socioeconomic status, race, and gender, may affect ownership of wearable fitness trackers.

## 2. Materials and Methods

### 2.1. Data Source and Study Population

This study used the All of Us Research Program Controlled Tier Dataset v8, accessed via the Researcher Workbench under an approved Data Use Agreement and in accordance with the program’s Data and Statistics Dissemination Policy. Data were extracted using Google BigQuery from within the secure Workbench environment.

The descriptive sample comprised all participants with complete demographic information (*n* = 633,547). The analytic sample for socioeconomic-adjusted models was restricted to the 606,414 participants with non-suppressed ZIP3 socioeconomic linkages, as detailed below.

### 2.2. Variable Construction

Demographics: Gender, race, ethnicity, sex at birth, and date of birth were extracted from the person table, with concept IDs joined to the concept table for labels. Age was calculated from date of birth using yrdif. For modeling, race was collapsed into 8 categories (White as reference), gender into 6 categories (Male as reference), and Hispanic/Latino ethnicity into 3 categories (Not Hispanic as reference). Small categories were combined as needed to satisfy the All of Us small-cell reporting threshold (*n* ≥ 20; see Table 1).

Socioeconomic indicators: ZIP3-level socioeconomic variables were linked to participants via the observation table. Six area-level indicators were initially extracted: median household income and fractions on assisted income, with a high school diploma, without health insurance, in poverty, and in vacant housing. The area-level deprivation index and assisted income were excluded due to high collinearity. The five retained continuous predictors (median household income; fractions with a high school diploma, without health insurance, in poverty, and in vacant housing) were rescaled for interpretable odds ratios (median income per USD 10,000; fractions per 10 percentage points).

Outcome: Fitbit ownership (San Francisco, CA, USA) (has_fitbit) was coded as 1 for participants with any record in the device table and 0 otherwise.

### 2.3. Missing Data and Cohort Construction

Missingness analysis was necessary due to the amount of missing results. Inspection of the source data confirmed that affected participants shared a single placeholder ZIP3 value not present in the socioeconomic lookup table, consistent with the All of Us program’s geographic data protections for participants residing in tribal lands or other small-population geographies under 20,000 people.

Because this represents structural data unavailability rather than item non-response, multiple imputation would have functioned as cross-group extrapolation rather than recovery of unobserved values. We therefore restricted socioeconomic-adjusted models to the participants with available SES data and report demographic characteristics for the suppressed participants separately to maintain their representation in descriptive results.

### 2.4. Statistical Analysis

Analyses were conducted in SAS via the All of Us SAS Cloud Environment (SAS Analytics Pro via SAS Studio, SAS Institute Inc., Cary, NC, USA; see File S1). Descriptive statistics were generated with PROC MEANS and PROC FREQ, both overall and stratified by Fitbit ownership. To characterize the participants excluded from the socioeconomic-adjusted models, the same descriptive statistics were computed separately for the SES-suppressed (*n* = 27,133) and SES-available (*n* = 606,414) subsamples and are presented side by side in Table 2, allowing the two groups to be compared directly on demographic, ethnoracial, ethnicity, Fitbit ownership, and area-level socioeconomic characteristics and any selection-related differences to be assessed.

Four binary logistic regression models predicted Fitbit ownership: (1) a demographics-only model (age, gender, race, and Hispanic/Latino ethnicity); (2) a ZIP3 socioeconomic-only model comprising the five retained area-level indicators; (3) a combined model including all demographic and socioeconomic predictors plus four median household income interaction terms (race × median household income, gender × median household income, age × median household income, and Hispanic/Latino ethnicity × median household income), to test whether the association between ZIP3 median household income and Fitbit ownership varied across each demographic dimension; and (4) an intersectional model that extended the Model 3 specification by adding a race × gender interaction, to test whether the gender difference in Fitbit ownership varied across racial groups. The unified focus on median household income as the SES moderator in Models 3 and 4 was retained for interpretive consistency (see Table 3, Table 4 and Table 5).

Median household income was centered at USD 65,000 before estimating the interaction models, since the interaction terms make the lower-order coefficients conditional on its value. This figure approximates the cohort mean (USD 64,944); uncentered, the demographic main effects and simple-effect odds ratios would be evaluated at an income of USD 0, which is not interpretable. Conditional odds ratios, including the female-versus-male odds ratios within each race group in Model 4 were computed at this mean. In Model 4, several race × gender strata were omitted from Table 6 because of quasi-separation or sparse cells (the Refused gender category, and transgender and non-binary participants within smaller race groups); stable estimates for these groups appear in Models 1 and 3.

Diagnostic analyses included pairwise Pearson correlations and variance inflation factors to inform selection of the final SES predictor set, distributional and box-plot examination of continuous variables for outliers (no observations were excluded on this basis), logistic regression of missingness on demographics to characterize the missing-data mechanism, and source-table inspection of ZIP3 response patterns to verify the suppression mechanism.

### 2.5. Ethical Considerations

The analysis was conducted using de-identified or generalized, publicly available data from the NIH All of Us Research Program. The All of Us program operates its own IRB, which approved the protocol, informed consent, and participant-facing materials; in accordance with NIH policies, our institution maintains a Data Use and Registration Agreement with the program. The present analysis was therefore determined exempt from institutional review board oversight at our institution.

## 3. Results

### 3.1. Participant Characteristics

The descriptive sample comprised 633,547 participants from the All of Us Controlled Tier Dataset (v8). Among these, 52,860 (8.34%) had at least one recorded Fitbit device, while 580,687 (91.66%) had no Fitbit record. The overall mean age was 56.5 years (SD = 17.1); Fitbit owners were marginally older (mean = 57.1 years) than non-owners (mean = 56.5 years). Fitbit owners resided in ZIP3 areas with slightly higher median household income (USD 65,999 vs. USD 64,848), higher fractions with high school education (88.4% vs. 87.0%), and lower fractions in poverty (14.5% vs. 15.7%) than non-owners (Table 1).

**Table 1:** Sample characteristics, overall and by Fitbit ownership. Continuous variables reported as mean ± SD, categorical variables reported as *n*, %.

Variable	Overall (*n* = 633,547)	Fitbit Owners (*n* = 52,860)	Non-Owners (*n* = 580,687)
Age (years)	56.54 ± 17.14	57.07 ± 16.37	56.50 ± 17.20
Fraction-Assisted Income (%)	14.46 ± 6.10	13.33 ± 5.46	14.56 ± 6.15
Fraction High School Education (%)	87.16 ± 5.59	88.41 ± 4.90	87.05 ± 5.64
Median Household Income (USD)	64,944 ± 16,829	65,999 ± 17,302	64,848 ± 16,782
Fraction No Health Insurance (%)	9.69 ± 4.21	9.07 ± 3.90	9.75 ± 4.23
Fraction Poverty (%)	15.56 ± 5.40	14.50 ± 5.17	15.66 ± 5.41
Fraction Vacant Housing (%)	10.06 ± 4.62	9.75 ± 4.91	10.09 ± 4.59
Gender			
Male	229,131 (36.17%)	16,589 (31.38%)	212,542 (36.60%)
Female	390,810 (61.69%)	34,876 (65.98%)	355,934 (61.30%)
Non-Binary	2886 (0.46%)	468 (0.89%)	2418 (0.42%)
Transgender	1173 (0.19%)	122 (0.23%)	1051 (0.18%)
Other Gender Identity	4018 (0.63%)	634 (1.20%)	3384 (0.58%)
Refused/Declined	5529 (0.87%)	171 (0.32%)	5358 (0.92%)
Race			
White	357,658 (56.45%)	36,850 (69.71%)	320,808 (55.25%)
Black or African American	99,788 (15.75%)	4088 (7.73%)	95,700 (16.48%)
None Indicated	91,621 (14.46%)	3947 (7.47%)	87,674 (15.10%)
More Than One Race	30,963 (4.89%)	3613 (6.84%)	27,350 (4.71%)
Asian	22,400 (3.54%)	2511 (4.75%)	19,889 (3.43%)
American Indian or Alaska Native	8973 (1.42%)	382 (0.72%)	8591 (1.48%)
NHPI/MENA (Combined)	4326 (0.68%)	363 (0.69%)	3963 (0.68%)
Refused/Declined	17,818 (2.81%)	1106 (2.09%)	16,712 (2.88%)
Ethnicity			
Not Hispanic or Latino	502,963 (79.39%)	45,412 (85.91%)	457,551 (78.79%)
Hispanic or Latino	112,751 (17.80%)	6342 (12.00%)	106,409 (18.32%)
Unknown/Declined	17,833 (2.81%)	1106 (2.09%)	16,727 (2.88%)

Of the cohort, 61.7% identified as female, 36.2% as male, and 2.1% as a gender minority or declined to answer. Self-reported race was White: 56.5%, Black or African American: 15.8%, Non- Indicated: 14.5%, more than one race: 4.9%, Asian: 3.5%, American Indian or Alaska Native (AIAN): 1.4%, Native Hawaiian/Pacific Islander or Middle Eastern/North African combined (NHPI/MENA): 0.7%, and 2.8% in non-response categories. Hispanic or Latino ethnicity was reported by 17.8% of participants. When examining Fitbit ownership rates within racial groups, Asian participants had the highest rate (11.2%), followed by more than one race (11.7%) and White (10.3%); Black or African American participants had the lowest among well-represented groups (4.1%).

### 3.2. Cohort Construction and Missing ZIP3 Socioeconomic Data

ZIP3-linked socioeconomic data were unavailable for 27,133 participants (4.28%), and the SES-adjusted regression models were therefore fitted on the remaining 606,414 participants. Missingness was strongly non-random and concentrated in two race groups: 100% of AIAN participants (n = 8973) and 57.4% of participants reporting more than one race (17,760 of 30,963), together accounting for 98.5% of all missingness. Demographic and Fitbit ownership characteristics for the 27,133 SES-suppressed vs. non-suppressed participants are reported in Table 2; Fitbit ownership in this group (8.61%) was comparable to the analytic cohort overall (8.33%; see Table 2).

**Table 2:** Characteristics of the SES-suppressed (n = 27,133) vs. non-suppressed (n = 606,414) cohort. Categorical variables reported as n (%), continuous variables reported as mean ± SD.

Variable	SES-Suppressed Cohort (n = 27,133)	SES-Available Cohort (n = 606,414)
Race		
White	168 (0.62%)	357,490 (58.95%)
Black or African American	75 (0.28%)	99,713 (16.44%)
None Indicated	103 (0.38%)	91,518 (15.09%)
More Than One Race	17,760 (65.46%)	13,203 (2.18%)
Asian	≤20	22,388 (3.69%)
American Indian or Alaska Native	8973 (33.07%)	0 (0.0%)
NHPI/MENA	≤20	4309 (0.71%)
Refused/Declined	25 (0.09%)	17,793 (2.93%)
Gender		
Male	9594 (35.36%)	219,537 (36.20%)
Female	16,590 (61.14%)	374,220 (61.71%)
Non-Binary	232 (0.86%)	2654 (0.44%)
Transgender	87 (0.32%)	1086 (0.18%)
Other Gender Identity	322 (1.19%)	3696 (0.61%)
Refused/Declined	308 (1.14%)	5221 (0.86%)
Ethnicity		
Not Hispanic or Latino	21,480 (79.17%)	481,483 (79.40%)
Hispanic or Latino	5628 (20.74%)	107,123 (17.66%)
Unknown/Declined	25 (0.09%)	17,808 (2.94%)
Fitbit Ownership		
Yes	2335 (8.61%)	50,525 (8.33%)
No	24,798 (91.39%)	555,889 (91.67%)
Continuous variables, mean (SD)		
Age (years)	53.5 ± 15.7	56.7 ± 17.2
Median household income (USD)	Suppressed	64,943.8 ± 16,828.9
High school education (%)	Suppressed	87.2 ± 5.6
No health insurance (%)	Suppressed	9.7 ± 4.2
Poverty (%)	Suppressed	15.6 ± 5.4
Vacant housing (%)	Suppressed	10.1 ± 4.6
Assisted income (%)	Suppressed	14.5 ± 6.1

### 3.3. Logistic Models

#### 3.3.1. Model 1—Demographics

The demographic logistic regression model on the analytic cohort (*n* = 606,414) significantly improved fit over the intercept-only null (likelihood ratio χ^2^ = 8517.0, df = 14, *p* < 0.0001), with relatively low discriminatory capacity (c = 0.613). Age was a statistically significant but practically negligible predictor (OR = 1.002 per year, 95% CI: 1.001–1.002, *p* < 0.0001).

Compared to males, females had 26% higher odds of Fitbit ownership (OR = 1.255, 95% CI: 1.230–1.280, *p* < 0.0001). Participants identifying as non-binary had more than twice the odds (OR = 2.207), and those in the combined other gender category had similarly elevated odds (OR = 2.160). Transgender participants also had significantly higher odds (OR = 1.504); the Refused/Declined gender category had significantly lower odds (OR = 0.480).

Race was a strong predictor. Compared to White participants, Black or African American participants had substantially lower odds (OR = 0.381, 95% CI: 0.368–0.394, *p* < 0.0001), as did those reporting None-Indicated (OR = 0.320) and NHPI/MENA (OR = 0.824). Asian participants (OR = 1.133) and those reporting more than one race (OR = 1.248) had significantly higher odds. Hispanic or Latino ethnicity was associated with higher odds of Fitbit ownership (OR = 1.247 vs. Not Hispanic, *p* < 0.0001). Estimates for the Refused race and Unknown ethnicity categories were not interpretable due to quasi-separation. AIAN participants are not represented in this model, as all are excluded from the SES-available analytic cohort. Full estimates are reported in Table 3.

**Table 3:** Odds ratios for Model 1, demographics-only model (*n* = 606,414). NR = not reported due to quasi-separation between Race = Refused and Ethnicity = Unknown categories (cells overlapping at the parameter level). American Indian or Alaska Native participants are not represented in this model because all were excluded from the SES-available cohort. Likelihood ratio χ^2^ = 8517.0 (df = 14), *p* < 0.0001; c-statistic = 0.613. Dependent variable: Fitbit ownership.

Predictor	Odds Ratio	Lower 95% CI	Upper 95% CI	*p*-Value
Age (per year)	1.002	1.001	1.002	<0.0001
Gender (ref: male)				
Female	1.255	1.23	1.28	<0.0001
Non-binary	2.207	1.986	2.453	<0.0001
Transgender	1.504	1.237	1.829	<0.0001
Other gender identity	2.16	1.971	2.366	<0.0001
Refused/declined	0.48	0.41	0.561	<0.0001
Race (ref: White)				
Black or African American	0.381	0.368	0.394	<0.0001
None indicated	0.32	0.302	0.34	<0.0001
More than one race	1.248	1.183	1.316	<0.0001
Asian	1.133	1.085	1.184	<0.0001
NHPI/MENA	0.824	0.739	0.918	0.0005
Refused/declined	NR	NR	NR	NR
Ethnicity (ref: Not Hispanic or Latino)				
Hispanic or Latino	1.247	1.185	1.312	<0.0001
Unknown/declined	NR	NR	NR	NR

#### 3.3.2. Model 2—Socioeconomic Indicators

The socioeconomic model included five ZIP3-level indicators (median household income, fractions with a high school diploma, without health insurance, in poverty, and in vacant housing). The model was statistically significant (likelihood ratio χ^2^ = 4229.3, df = 5, *p* < 0.0001) but offered weaker discrimination than the demographic model (c = 0.589). Median household income was the dominant predictor: each USD 10,000 increase in ZIP3 median household income was associated with 12.5% lower odds of Fitbit ownership (OR = 0.875, 95% CI: 0.867–0.882, *p* < 0.0001). The remaining four area-level fractions were statistically significant but had negligible effect magnitudes (ORs of 0.996 to 1.004, all *p* ≤ 0.0004 except no_health_insurance *p* = 0.0004 with OR ≈ 1.001). Full estimates are reported in Table 4.

**Table 4:** Odds ratios for Model 2, ZIP3 socioeconomic indicators-only model (*n* = 606,414). χ^2^ = 4229.3 (df = 5), *p* < 0.0001; c-statistic = 0.589. Dependent variable: Fitbit ownership.

Predictor	Odds Ratio	Lower 95% CI	Upper 95% CI	*p*-Value
Median household income (per USD 10,000)	0.875	0.867	0.882	<0.0001
High school education (per 10 pp)	1.004	1.004	1.004	<0.0001
No health insurance (per 10 pp)	1.001	1	1.001	0.0004
Poverty (per 10 pp)	0.996	0.995	0.996	<0.0001
Vacant housing (per 10 pp)	0.998	0.998	0.998	<0.0001

#### 3.3.3. Model 3—Combined Model with Demographic × SES Interactions

The combined model incorporated all demographic and socioeconomic predictors plus four interaction terms, median household income × race, gender, age, and Hispanic/Latino ethnicity, to evaluate whether the income by Fitbit ownership association varied across major demographic dimensions. The combined model significantly improved over Models 1 and 2 individually (likelihood ratio χ^2^ = 10,342.7, df = 33, *p* < 0.0001), with a higher discrimination of the previous two models, though still relatively low (c = 0.630). Joint tests indicated that race × median income and gender × median income were significant moderators (both *p* < 0.0001), while age × median income (*p* = 0.09) and ethnicity × median income (*p* = 0.62) were not.

Many race × median income interactions were significant. Notably, for nearly all racial groups, residing in a higher-income ZIP3 was associated with lower Fitbit ownership odds. For Black participants, the relationship reversed direction, residing in a higher-income ZIP3 was associated with higher odds.

Gender × median income interactions were significant only for female compared to male, which showed females had a steeper negative income slope than males (female: OR = 0.874 vs. male reference OR = 0.927, joint *p* < 0.0001). All other gender categories showed similar negative slopes, though had non-significant interaction effects.

Age × median income interactions were non-significant, with income ORs nearly identical at 30 (OR = 0.921), 50 (OR = 0.927), and 70 (OR = 0.932) years of age, consistent with the non-significant joint test (*p* = 0.09).

Ethnicity × median income interactions were non-significant, with the Hispanic income OR (0.941, 95% CI: 0.911–0.972) comparable to the Not Hispanic reference (0.927); the joint test was non-significant (*p* = 0.62).

Main effects for the four SES variables not involved in interactions (high school education, no health insurance, poverty, vacant housing) remained statistically significant but small in magnitude (all |OR − 1| < 0.004). Full interaction-specific estimates are reported in Table 5.

**Table 5:** Model statistics for combined model (demographics + SES) with median income × demographic interactions (*n* = 606,414; LR χ^2^ = 10,342.7, df = 33, *p* < 0.0001; c = 0.630). Main effects shown for SES variables not in interactions; conditional odds ratios show the income effect per USD 10,000 increase at each moderator level (non-focal moderators fixed at reference). Race, gender, age, and ethnicity main effects are omitted because they correspond to median income = USD 0 and are not substantively interpretable. Quasi-separated categories (Race = Refused, Ethnicity = Unknown) omitted. Dependent variable = Fitbit ownership.

**Joint Tests of Effects**				
**Effect**	**df**	**Wald χ^2^**	***p*** **-Value**	
Gender	5	252.4	<0.0001	
Age	1	0.004	0.95	
Race	6	607.6	<0.0001	
Ethnicity	2	2.99	0.22	
High school education	1	519.3	<0.0001	
Median household income	1	49.8	<0.0001	
No health insurance	1	100.6	<0.0001	
Poverty	1	187.4	<0.0001	
Vacant housing	1	195.4	<0.0001	
Median income × Race	6	188.2	<0.0001	
Median income × Gender	5	99.9	<0.0001	
Age × Median income	1	2.79	0.09	
Median income × Ethnicity	2	0.95	0.62	
**Main Effects**				
**Predictor**	**Odds Ratio**	**Lower 95% CI**	**Upper 95% CI**	***p*** **-Value**
High school education (per 10% change)	1.004	1.003	1.004	<0.0001
No health insurance (per 10% change)	1.002	1.001	1.002	<0.0001
Poverty (per 10% change)	0.998	0.997	0.998	<0.0001
Vacant housing (per 10% change)	0.998	0.998	0.998	<0.0001
**Conditional Odds Ratios**				
**Moderator Level**	**Odds Ratio**	**Lower 95% CI**	**Upper 95% CI**	
By Race (at age = 50, male, Not Hispanic)				
White (reference)	0.927	0.915	0.939	
Black or African American	1.079	1.053	1.107	
None Indicated	0.925	0.889	0.963	
More than one race	0.963	0.933	0.995	
Asian	0.981	0.959	1.004	
NHPI/MENA	0.905	0.851	0.962	
By Gender (at age = 50, White, Not Hispanic)				
Male (reference)	0.927	0.915	0.939	
Female	0.874	0.864	0.884	
Non-binary	0.881	0.823	0.943	
Transgender	0.907	0.797	1.031	
Other gender identity	0.904	0.856	0.955	
Refused/declined	0.983	0.899	1.074	
By Age (at Male, White, Not Hispanic)				
Age 30	0.921	0.906	0.937	
Age 50 (reference)	0.927	0.915	0.939	
Age 70	0.932	0.921	0.943	
By Ethnicity (at age = 50, male, White)				
Not Hispanic (reference)	0.927	0.915	0.939	
Hispanic or Latino	0.941	0.911	0.972	

#### 3.3.4. Model 4—Intersectional Model with Race × Gender Interactions

Model 4 extended the Model 3 specification by adding a race × gender interaction to test whether the gender difference in Fitbit ownership varied across racial groups, with median household income centered at USD 65,000. It fit significantly better than the null (likelihood ratio χ^2^ = 10,814.5, df = 63, *p* < 0.0001) and showed the highest discrimination of any model, though still low (c = 0.632). The race × gender interaction was statistically significant (Wald χ^2^ = 440.9, df = 30, *p* < 0.0001).

Because of the large number of strata, we report only the female-versus-male odds of ownership within each race group (Table 6). The female advantage observed overall was not uniform across race. It was largest among Black or African American participants (OR = 2.265, 95% CI: 2.101–2.442, *p* < 0.0001) and also present among White (OR = 1.227, 95% CI: 1.199–1.257, *p* < 0.0001) and more than one race (OR = 1.560, 95% CI: 1.387–1.754, *p* < 0.0001) participants. Among Asian participants the direction reversed, with females showing lower odds than males (OR = 0.867, 95% CI: 0.796–0.946, *p* = 0.001). Differences were non-significant for None-Indicated (OR = 0.967, *p* = 0.34) and NHPI/MENA (OR = 0.940, *p* = 0.58) participants. The Refused gender category and the transgender and non-binary strata within smaller race groups were omitted because of quasi-separation or unstable estimates; stable estimates for these groups appear in Models 1 and 3.

**Table 6:** Race × gender interaction. Female-versus-male odds of Fitbit ownership within each race group, from a model that adds a race × gender interaction to the combined specification in Table 5 (n = 606,414; LR χ^2^ = 10,814.5, df = 63, *p* < 0.0001; c = 0.632). Median household income centered at USD 65,000. The race × gender interaction was statistically significant (Wald χ^2^ = 440.9, df = 30, *p* < 0.0001). Reference = male within each race group. Refused category and sparse race × gender strata (transgender and non-binary participants within smaller race groups) are omitted as quasi-separated or unstable. Dependent variable = Fitbit ownership.

INTERSECTIONAL OR—Female vs. Male, by Race (at Median Income = USD 65,000)						
	df	Wald χ^2^	*p*-Value	OR	Lower 95% CI	Upper 95% CI
Race × Gender (joint test)	30	440.9	<0.0001			
Female vs. Male, by race—White			<0.0001	1.227	1.199	1.257
Female vs. Male, by race—Black			<0.0001	2.265	2.101	2.442
Female vs. Male, by race—None Indicated			0.344	0.967	0.903	1.035
Female vs. Male, by race—More than one race			<0.0001	1.560	1.387	1.754
Female vs. Male, by race—Asian			0.001	0.867	0.796	0.946
Female vs. Male, by race—NHPI/MENA			0.575	0.940	0.754	1.170

## 4. Discussion

In this analysis of wearable device ownership using the All of Us Research Program dataset, several notable patterns emerged regarding demographic and socioeconomic predictors. Of particular interest, our findings are in line with previously published literature, in regards to gender and race device ownership rates, while providing a more nuanced socioeconomic picture: ZIP3-level area income was a substantial predictor of wearable ownership in this cohort, and its effect varied significantly across racial groups in ways not previously documented.

Prior consumer-driven studies have highlighted pronounced individual-level socioeconomic disparities in wearable ownership, reflecting the economic burden of device acquisition [7,11]. The present analysis used data from a cohort enriched by the federally funded WEAR initiative, in which a substantial proportion of participants received devices free of charge. This design partially reduces individual-level financial barriers, but our findings indicate that area-level socioeconomic context continues to shape wearable ownership even in a cohort with subsidized device access. Increases in ZIP3-level median household income were associated with lower odds of Fitbit ownership (OR = 0.875, 95% CI: 0.867–0.882). This counterintuitive negative gradient, opposite to what consumer-driven studies typically report, may be evidence that the device distribution disproportionately reached lower-income participants, which would be a boon to researchers, and/or this is showing evidence of a difference between wearable technology ownership rates among socioeconomic populations.

### 4.1. Demographic—Gender

Our results identified distinct gender-based patterns in wearable ownership. Consistent with previous studies, female participants exhibited 1.26-times-higher odds of owning wearable devices compared to male counterparts (based on demographics only model). This finding closely mirrors previous research, which also reported women as approximately 1.2 to 1.3 times more likely than men to own and use wearable technology [11]. Additionally, a notable contribution of this research is the significantly increased wearable ownership among gender-diverse individuals. Participants identifying as non-binary (OR = 2.207), transgender (OR = 1.504), or in the combined other gender Identity category (OR = 2.160) exhibited significantly greater odds of owning a wearable compared to male participants. The inclusion of these diverse gender categories is an important strength of the All of Us dataset, reducing the gender limitations of other datasets, which often provide only binary gender options, consequently limiting generalizability and potentially excluding gender-diverse individuals. Gender moderation of the income effect was observed: female participants showed a steeper negative income gradient (OR = 0.874 per USD 10,000) than male participants (OR = 0.927), suggesting that the interaction effect of area income on wearable ownership is somewhat amplified for women.

### 4.2. Demographic—Ethnoracial

In the present analysis, we found modest racial differences in wearable ownership: Asian (OR = 1.133) and more than one race (OR = 1.248) participants had higher odds of Fitbit ownership compared to White participants, while Black or African American (OR = 0.381), None-Indicated (OR = 0.320), and NHPI/MENA (OR = 0.824) participants had lower odds (based on demographics only model). American Indian or Alaska Native participants could not be included in adjusted models due to structural unavailability of their ZIP3-linked socioeconomic data.

Hispanic or Latino ethnicity, captured independently from race in the *All of Us* Basics survey, was significantly associated with higher odds of Fitbit ownership compared to non-Hispanic participants (OR = 1.247, 95% CI: 1.185–1.312, *p* < 0.0001). This positive association is a dimension that studies combining race and ethnicity into a single variable may obscure. Notably, the area-level income gradient did not differ significantly between Hispanic and non-Hispanic participants (interaction *p* = 0.62), suggesting that socioeconomic context shapes wearable ownership similarly across ethnic groups.

These findings complement earlier literature, which indicated similar trends among children [12] and adults [7]. The detailed racial categorization enhances the robustness of our analysis and provides nuanced insights compared to prior literature, which often employs narrower or less inclusive racial definitions. However, the persistently lower wearable ownership among Black participants, American Indian or Alaska Native, underscores ongoing disparities and highlights the need for targeted, culturally sensitive engagement strategies to improve equitable access and ownership.

### 4.3. Socioeconomic—Income Interaction

An important novel contribution of this analysis is the identification of a significant race × median income interaction. While earlier research, such as Nagappan et al.’s, has documented strong correlations between wearable ownership and higher socioeconomic status (SES), including higher income, educational attainment, and employment status [7,13], our results show that the All of Us dataset does not follow that same trend.

For nearly all racial groups, residing in a higher-income ZIP3 was associated with **lower odds of Fitbit ownership**. For Black or African American participants, however, this relationship reversed direction: higher area-level income was associated with **higher odds of Fitbit ownership**.

This opposite-direction gradient suggests that the contextual mechanisms linking neighborhood socioeconomic conditions to wearable ownership may operate differently for Black participants than for other groups. One possibility is sampling variability within different populations, though the large All of Us sample size attenuates this concern. A more probable source is device coverage: Nagappan et al. captured ownership of any wearable, so Black respondents owning non-Fitbit devices such as Apple Watches would be counted as owners there but not in our Fitbit-only analysis. The WEAR provisioning pathway likely also contributes, since it directed free devices toward lower-socioeconomic and underrepresented communities, which would raise Fitbit ownership among lower-income Black participants and could produce the observed positive income relationship in this group. This finding warrants targeted follow-up in future studies.

### 4.4. Limitations

A significant limitation of our socioeconomic analyses is that ZIP3-linked socioeconomic data were structurally unavailable for all American Indian or Alaska Native participants (n = 8973) and for approximately 57% of participants reporting more than one race (n = 17,760), reflecting the *All of Us* program’s ZIP3 aggregation policy for areas with fewer than 20,000 participants. Because these populations are disproportionately resident in lower-population (including tribal land) ZIP3 areas, the aggregation rule produces a structural data gap that disproportionately affects historically underrepresented groups. Consequently, our socioeconomic-adjusted models cannot generate estimates for AIAN participants, and estimates for the multiple race category reflect only the non-AIAN-overlap subset. We report demographics and Fitbit ownership rates for these participants descriptively, but their absence from the regression models is itself a limitation to the current analysis.

As the sample size for this population-level analysis is large, results (whether significant or not) should be viewed in connection with the effect sizes (odds ratios) to determine the magnitude of any differences that exist between groups. Additionally, our socioeconomic indicators were assessed at the ZIP3 area level, capturing neighborhood context but masking individual-level economic variation. Observed area-level associations may not reflect individual-level economic effects (ecological inference limitation).

Although the All of Us dataset included expansive gender identity options, it failed to distinguish between transgender male and transgender female identities, with the only option available for selection as “transgender”. Such categorical limitations might obscure meaningful differences within this demographic group. Moreover, while our dataset includes a higher proportion of female participants, which allows for robust analysis of historically underrepresented groups in biomedical research, it may simultaneously limit the generalizability of our findings to populations with more balanced gender distributions.

A further limitation concerns the device-acquisition pathway. The All of Us dataset, to our knowledge, contains no identifier distinguishing Fitbits provisioned through the WEAR initiative from those self-acquired and contributed through the Bring-Your-Own-Device pathway, so we could not refit models by pathway or run a sensitivity analysis. Because these pathways likely relate to socioeconomic status differently, the negative income gradient and its reversal among Black or African American participants may partly reflect their mix rather than ownership behavior alone. Additionally, our analysis utilized data specifically from Fitbit devices, restricting the findings to ownership and initial synchronization rather than sustained use or data quality. This could limit conclusions regarding long-term wearable engagement (which was not the focus of this investigation). The limitation to only Fitbit ownership may explain the negative ownership rates by median income categories, as those of higher socioeconomic backgrounds may adopt a different wearable device at higher rates (for example Apple Watches).

This dataset’s comprehensive participant base and the method of wearable distribution ensure its utility for broad population-level research. Unlike previous research, such as the UK-based study by Strain et al. [14], which cautioned that wearable data primarily represents younger, more active, and affluent individuals, our findings suggest that the All of Us dataset, at least partially, mitigates these biases, particularly among age concerns with the average age of 57 years among the Fitbit population, though direct database comparisons are warranted to further elucidate the mitigation the All of Us dataset may provide. Despite lacking direct comparisons, this resource likely represents a more equitable data repository for population-level studies of physical activity and related health outcomes.

Practically, this dataset can serve as a foundational resource for health researchers, policymakers, and public health professionals aiming to design and implement interventions to improve health outcomes across diverse populations. For example, the equitable distribution and minimal socioeconomic bias of wearable data in the All of Us dataset can facilitate targeted interventions addressing physical activity disparities among underrepresented and economically disadvantaged groups. Additionally, public health programs can leverage insights into gender and racial disparities to create more inclusive and culturally competent health promotion initiatives. Overall, these data offer valuable insights to help bridge gaps in wearable technology ownership, ultimately enhancing the effectiveness and reach of public health interventions and research efforts.

## 5. Conclusions

In conclusion, this analysis demonstrates that wearable device ownership in the *All of Us* cohort is shaped by both individual demographic identity and area-level socioeconomic factors. Female participants, gender-diverse individuals, and Hispanic or Latino participants had higher odds of Fitbit ownership, while Black or African American, NHPI/MENA, and other underrepresented racial groups had lower odds. Area-level median household income was a meaningful predictor of ownership, but the direction and magnitude of this effect varied by race, most notably, the income–ownership relationship reversed direction for Black or African American participants compared to all other groups. Together, these findings reinforce the necessity for inclusive demographic capture, explicit testing of demographic moderation in socioeconomic models, and continued attention to structural data gaps that disproportionately affect historically underrepresented populations. Future research should expand beyond initial device ownership to sustained engagement metrics and examine intersectional moderators that may further inform equitable wearable distribution and research strategies.

## Data Availability

The SAS syntax used for this analysis has been provided as a Supplementary File. Dataset used is a public dataset maintained by NIH as part of the All of Us Research Program.

## Supplementary Materials

The following supporting information can be downloaded at: https://www.mdpi.com/article/10.3390/ijerph23070839/s1, File S1: The SAS syntax.

## References

[1] Iqbal M.H., Aydin A., Brunckhorst O., Dasgupta P., Ahmed K. (2016). A review of wearable technology in medicine. J. R. Soc. Med..

[2] Bunn J.A., Navalta J.W., Fountaine C.J., Reece J.D. (2018). Current State of Commercial Wearable Technology in Physical Activity Monitoring 2015-2017. Int. J. Exerc. Sci..

[3] Xie J., Wen D., Liang L., Jia Y., Gao L., Lei J. (2018). Evaluating the Validity of Current Mainstream Wearable Devices in Fitness Tracking Under Various Physical Activities: Comparative Study. JMIR mHealth uHealth.

[4] Thompson W.R. (2016). Worldwide Survey of Fitness Trends for 2017. ACSM’s Health Fit. J..

[5] Newsome A.M., Batrakoulis A., Camhi S.M., Mcavoy C., Sansone J., Reed R. (2024). 2025 ACSM Worldwide Fitness Trends: Future Directions of the Health and Fitness Industry. ACSM’s Health Fit. J..

[6] Beckett D., Curtis R., Szeto K., Maher C. (2025). Changing User Experience of Wearable Activity Monitors Over 7 Years: Repeat Cross-Sectional Survey Study. J. Med. Internet Res..

[7] Nagappan A., Krasniansky A., Knowles M. (2024). Patterns of Ownership and Usage of Wearable Devices in the United States, 2020-2022: Survey Study. J. Med. Internet Res..

[8] Cheung M.L., Chau K.Y., Lam M.H.S., Tse G., Ho K.Y., Flint S.W., Broom D.R., Tso E.K.H., Lee K.Y. (2019). Examining Consumers’ Adoption of Wearable Healthcare Technology: The Role of Health Attributes. Int. J. Environ. Res. Public Health.

[9] Denny J.C., Rutter J.L., Goldstein D.B., Philippakis A., Smoller J.W., Jenkins G., Dishman E., All of Us Research Program (2019). The “All of Us” Research Program. N. Engl. J. Med..

[10] All of Us Research Program News and Events Research Roundup: All of Us Participants’ Fitbit Data Drive New Research. https://allofus.nih.gov/article/announcement-research-roundup-all-us-participants-fitbit-data-drive-new-research.

[11] Chandrasekaran R., Katthula V., Moustakas E. (2020). Patterns of Use and Key Predictors for the Use of Wearable Health Care Devices by US Adults: Insights from a National Survey. J. Med. Internet Res..

[12] Kim E.H., Jenness J.L., Miller A.B., Halabi R., de Zambotti M., Bagot K.S., Baker F.C., Pratap A. (2023). Association of Demographic and Socioeconomic Indicators with the Use of Wearable Devices Among Children. JAMA Netw. Open.

[13] Shandhi M.M.H., Singh K., Janson N., Ashar P., Singh G., Lu B., Hillygus D.S., Maddocks J.M., Dunn J.P. (2024). Assessment of ownership of smart devices and the acceptability of digital health data sharing. npj Digit. Med..

[14] Strain T., Wijndaele K., Brage S. (2019). Physical activity surveillance through smartphone apps and wearable trackers: Examining the UK potential for nationally representative sampling. JMIR mHealth uHealth.

